# Assessing the role of variety renewal in enhancing agro-product regional public brand value: a case study of Gaizhou grapes

**DOI:** 10.3389/fpls.2026.1877640

**Published:** 2026-07-09

**Authors:** Ruilong Zhang, Jutao Zeng, Xiaoli Yang

**Affiliations:** 1 School of Economics and Management, Liaoning University of Traditional Chinese Medicine, Shenyang, China; 2 College of Economics and Management, Shenyang Agricultural University, Shenyang, China

**Keywords:** agro-product regional public brand, farmer income, rural development, sustainable horticulture, variety renewal

## Abstract

**Introduction:**

Analyzing the stability of agro-product regional public brands from the perspective of variety renewal can address the shortcomings of existing micro-level research. Using Gaizhou grapes in Liaoning Province, China, as a case study, this study examines the large-scale replacement of the traditional Kyoho grape with Shine Muscat and explores how variety renewal affects farmers’ production and economic outcomes.

**Methods:**

Ordinary least squares (OLS), instrumental variable (IV), and quantile regression models were employed to estimate the effects of variety renewal on grape yield, income, and unit prices. The average value of variety renewal choices among other farmers in the same village was used as an instrumental variable to address potential endogeneity.

**Results:**

The results show that variety renewal reduces grape yield but significantly increases farmers’ total income and unit prices. The IV estimates confirm the robustness of these findings. The quantile regression results reveal substantial heterogeneity in the effects of variety renewal across renewal patterns and different points of the yield, revenue, and unit price distributions. Full renewal is more likely to improve revenue and unit prices among farmers at higher revenue or quality levels, whereas its yield effects are less favorable among farmers with medium or lower yield levels. By contrast, partial renewal tends to generate weaker economic gains and may even reduce yield or revenue among farmers at medium outcome levels.

**Discussion:**

Rising production costs are an important factor contributing to the divergence between yield and income outcomes. Farmer training, improved access to information, enhanced online sales capability, stronger social learning, and greater social capital can mitigate yield losses associated with variety renewal to varying degrees, although their moderating effects differ across farm-scale groups. Overall, the findings indicate that variety renewal can enhance the value of regional agricultural products and support farmers’ income growth, but its benefits depend on the degree of renewal, farm scale, and farmers’ capabilities. This study provides empirical evidence for promoting agricultural brand upgrading and designing differentiated strategies for sustainable rural income growth.

## 1 Introduction

Agricultural branding has always been a crucial component of agricultural development, helping to enhance the market value of agricultural products (Chaturvedi and Sinha, 2024). Regional public brands for agricultural products are an integral part of agricultural branding and play a significant role in increasing farmers’ incomes (Wang et al., 2022; Tang et al., 2024). The Chinese government places great emphasis on agricultural brand building, and in recent years, the annual Central Document No.1 has consistently included directives related to brand development. For example, the 2017 Central Document No.1 proposed “advancing the development of regional public brands of agricultural products and supporting localities in creating regionally distinctive brands based on leading enterprises and industry associations”; the 2020 Document called for “developing well-known local agricultural product brands and increasing the supply of high-quality, green agricultural products”; the 2023 Document proposed “supporting poverty-stricken areas in developing regional public brands”; and the 2025 and 2026 Documents both mentioned plans for cultivating high-quality agricultural brands. From a policy perspective, agricultural brand development has shifted from a focus on “how to expand and strengthen” to “how to achieve higher-quality, premium development,” and the same applies to regional public brands of agricultural products.

However, most existing literature focuses on the factors influencing the growth and development of regional agricultural public brands (Wiedmann et al., 2011; Xu et al., 2024), while empirical research on the higher-quality upgrading and development of agricultural brands remains scarce. In particular, as agricultural brands with clearly defined production areas, regional public agricultural brands involve numerous agricultural suppliers and possess unique characteristics that directly impact the income levels of ordinary farmers (Zheng and Li, 2024). Existing studies have shown that Geographical Indication (GI) certification is an important means of establishing regional public brands (Su, 2013), and GI-certified agricultural products can promote agricultural development by protecting origin-specific quality, preserving related technologies and knowledge, enhancing producers’ technical capabilities, and strengthening market competitiveness (Lawal-Arowolo, 2019; Li et al., 2024). GI agricultural products are also more likely to gain consumer trust (Iraizoz et al., 2011). A Regional Public Brand of Agricultural Products (RPBAP) refers to a brand owned by relevant organizations and jointly used by multiple agricultural production and management entities within a specific production area characterized by unique natural ecosystems, historical backgrounds, and cultural features (Zheng and Li, 2024). By leveraging brand advantages, it influences consumers’ attitudes toward individual enterprise brands, improves brand image, or reinforces brand characteristics, thereby promoting consumer purchase intent (Cao, 2011), increase farmers’ bargaining power, and promote the added value of agricultural products and industrial development (Ohe and Kurihara, 2013; Yin et al., 2024). Consistent with these findings, studies based on Chinese agricultural products have shown that, in China, agricultural GIs and regional agricultural brands have been linked to county-level economic growth, agricultural export performance, and changes in farmers’ income distribution (Qie et al., 2023; Zhang et al., 2023; Zheng and Li, 2024). However, their contribution to agrifood quality improvement is not automatic. Zhao et al. (2014) found that some Chinese GI schemes were constrained by low standards, inappropriate issuing procedures, and weak government inspection, suggesting that certification alone may be insufficient to enhance product quality. In terms of influencing factors, the development of regional public agricultural brands depends on market maintenance, public marketing, industrial resources, regional resources, and institutional capability (Wiedmann et al., 2011; Xu et al., 2019, Xu et al., 2024). Subsequent empirical and qualitative studies have also emphasized the role of government institutional capability and multi-actor governance in regional brand development (Lei, 2015; Zhao and Sun, 2017; Qi et al., 2021; Zhang et al., 2022; Li et al., 2022). Nevertheless, these studies mainly examine brand construction, institutional governance, consumer response, or regional economic effects, while paying insufficient attention to the production-side mechanisms through which regional public agricultural brands are upgraded and sustained. For regional public agricultural brands, long-term brand value depends not only on institutional support and public marketing, but also on continuous product quality improvement, stable market supply, and farmers’ ability to adapt to changes in consumer demand.

In the context of China’s current agricultural transformation, crop variety renewal has emerged as one of the pathways to drive the upgrading of regional agricultural products. In particular, variety renewal directly affects crop yields and productivity (Evenson and Gollin, 2003; Hansen et al., 2006; Yu et al., 2012), and may also improve product appearance, taste, storability, market recognition, and price premiums. However, variety renewal also brings uncertainty to both production and markets. On the one hand, introducing new varieties into production requires farmers to navigate a steep learning curve (Atlin et al., 2017), and may involve higher input intensity, additional management requirements, and rising production costs. According to producer theory, increased costs may reduce output under certain production conditions (Mankiw, 2021), thereby threatening market supply levels. On the other hand, differences may exist between producers’ expectations and consumers’ willingness to pay for new agricultural products, and new products may face a lukewarm market response (Kilduff and Tregeagle, 2022). This issue is particularly relevant to the table grape industry. Studies on Chinese grape growers show that cultivar adoption is shaped by varietal attributes, featured consumption attributes, and risk-control management (Wang et al., 2017). Research on China’s table grape supply chains further indicates that safety and quality control, supply-chain organization, and value-chain performance affect the production outcomes of small-scale grape growers (Jiao et al., 2012; Deng et al., 2016). In the table grape sector, protected cultivation has become an important support for production upgrading; however, studies on protected grape cultivation in China indicate that such systems involve complex resource-input structures, facility-related materials, and technical management requirements (Feng et al., 2015; Tian et al., 2018). Therefore, although variety renewal may enhance the value of regional agricultural products by improving quality and generating higher prices, its effects on farmers’ yield, income, and unit prices remain theoretically ambiguous. Existing studies have examined regional agricultural branding, GI certification, grape cultivar adoption, supply-chain performance, and protected grape cultivation, but these strands of literature have rarely been integrated to explain how variety renewal functions as a production-side mechanism for upgrading regional public agricultural brands. Moreover, little is known about whether the economic consequences of full and partial variety renewal differ across farm scales. These gaps provide the motivation for examining the replacement of Kyoho grapes with Shine Muscat in Gaizhou.

This paper uses detailed case-based data to examine how variety renewal can support the high-quality development of regional public brands of agricultural products. Drawing on the research project and accumulated fieldwork experience, the research team has established close cooperative relationships with relevant departments in Gaizhou City, Liaoning Province. The “renewal and innovation” of Gaizhou grapes provides a solid case study for the aforementioned research. Gaizhou City is one of the typical grape-producing regions in Liaoning, with an annual output of approximately 300,000 tons, making it highly representative. As a regional public brand, Gaizhou City Grapes encompasses multiple grape varieties, such as Kyoho, Liaofeng, and Rose Fragrance, exhibiting brand characteristics similar to an “umbrella brand” (Iversen and Hem, 2008). However, unlike an “umbrella brand,” Kyoho has long been the dominant grape variety in Gaizhou, accounting for a large share of local grape production and making it highly representative of the regional industry. In recent years, the Gaizhou grape industry has been undergoing an upgrade driven by variety renewal. To meet market demand, many growers have switched to planting Shine Muscat grapes. Field surveys reveal that the vast majority of growers who switched to Shine Muscat previously cultivated Kyoho grapes. From a county-wide perspective, the primary cultivated variety of Gaizhou City Grapes is shifting from Kyoho to Shine Muscat. These realities provide a suitable case study for researching the impact of variety renewal on the development of regional public brands of agricultural products.

Based on the above data, this paper examines the relationship between variety renewal and the development of regional public brands of agricultural products. Variety renewal is categorized into two dimensions: whether replacement occurred (a binary variable) and the extent of replacement (complete replacement, partial replacement, or no replacement). The development of regional public brands of agricultural products is measured by three indicators: grape production, revenue, and unit price. This paper first empirically examines the extent to which variety renewal affects yield, revenue, and unit price, and addresses the issue of endogeneity by using the average value of variety renewal choices among other farmers in the same village as an instrumental variable. Subsequently, this study employs quantile regression to examine the further effects of the degree of replacement on yield, revenue, and unit price, and analyzes the underlying mechanisms through which variety renewal influences brand development using chemical fertilizer application and farmyard manure application as variables reflecting cost changes. Finally, this study further explores the heterogeneity of variety renewal from five perspectives: training, information access capability, online sales capability, social learning, social capital networks.

The study found that: First, variety renewal led to a decline in grape yield, but total revenue and unit prices increased significantly. Second, the effects of variety renewal on grape yield, revenue, and unit price exhibit substantial heterogeneity across renewal patterns and different quantiles of the outcome distributions. Specifically, full renewal and partial renewal have markedly different effects on farmers’ production and economic outcomes. Full renewal tends to reduce yield among farmers with medium or lower yield levels, but it significantly increases revenue among farmers with medium or higher revenue levels and raises unit prices for farmers with higher-quality output. By contrast, partial renewal mainly leads to yield declines among farmers with medium yield levels, reduces revenue among farmers with medium and relatively higher revenue levels, and has no significant effect on unit price. These results suggest that the effects of variety renewal depend on farmers’ relative positions in the yield, revenue, and unit price distributions. Third, cost changes associated with variety renewal partly explain the observed differences in yield and income outcomes. Fourth, regarding heterogeneity, measures such as expanding training, improving information technology capabilities, enhancing online sales capability, strengthening social learning abilities, and increasing social capital can all mitigate, to varying degrees, the yield declines caused by variety renewal.

The academic contributions of this study are threefold. First, this study extends the literature on regional public brands of agricultural products by introducing variety renewal as a production-side mechanism of brand upgrading. Previous studies on product renewal have mainly focused on industrial and commercial products (Yin et al., 2010; Jung et al., 2022; Okada, 2001, Okada, 2006; Bellezza et al., 2017; Dagogo-Jack and Forehand, 2018; Zhu et al., 2008). However, agricultural variety renewal differs from industrial product renewal because it is embedded in biological production processes, seasonal constraints, farmers’ technical learning, production costs, and market uncertainty. By focusing on the replacement of Kyoho grapes with Shine Muscat in Gaizhou, this study shifts the discussion of product renewal from firm-level product strategy to the production-side upgrading of regional public agricultural brands. Second, this study enriches research on agricultural variety renewal and table grape industry upgrading. Existing studies on crop varieties have mainly focused on yield, productivity, cultivar adoption, or technical preferences, while studies on China’s table grape industry have emphasized supply-chain organization, quality control, and protected cultivation. This study links these perspectives by examining whether variety renewal affects not only yield but also farmers’ income and unit prices, thereby clarifying the cost–benefit trade-offs associated with fruit variety renewal under regional brand development. More specifically, this study embeds variety renewal within the theories of returns to scale and cost–benefit analysis, while incorporating the characteristics of horticultural crop renewal and the spillover effects of regional public brands. By empirically examining the effects of variety renewal on production capability, market returns, and farmers’ income, this study identifies the underlying logic through which variety renewal may enhance the value of regional public agricultural brands. At the economic level, variety renewal may improve the quality and efficiency of regional agricultural industries by generating higher market premiums and increasing farmers’ income; at the social level, it may support rural industrial revitalization and the construction of regional agricultural brand systems. Through benchmark regressions and quantile regression analysis, this study further clarifies the intrinsic logic and transmission mechanisms linking variety renewal to the upgrading of regional public agricultural brands. Third, this study provides farm-level evidence on the heterogeneous effects of variety renewal. By distinguishing full and partial variety renewal and examining how their effects vary across different points of the yield, revenue, and unit price distributions, this study identifies the boundary conditions under which variety renewal enhances or weakens farmers’ economic returns. In addition, by considering production costs, farmer training, information access capability, online sales capability, social learning, and social capital networks, this study further explains why the benefits of variety renewal are not evenly distributed among farmers. These findings contribute to a more nuanced understanding of how regional public agricultural brands can achieve high-quality upgrading while supporting sustainable rural income growth.

The structure of this paper is as follows: Part One is the Introduction; Part Two covers Materials and Methods, presenting the theoretical framework and describing the data; Part Three empirically examines whether variety renewal occurs and the extent to which it affects the development of regional public brands of agricultural products; Part Four analyzes heterogeneity to examine how the impact of variety renewal on grape yield varies under different constraints; and Part Five summarizes the findings and offers corresponding policy recommendations.

## 2 Materials and methods

### 2.1 Theoretical framework

Based on the theories of returns to scale and cost–benefit analysis, this study incorporates the characteristics of horticultural crop variety renewal and the spillover effects of regional public brands to develop an analytical framework. Through mathematical derivation, the framework explains how grape variety renewal may affect the value enhancement of regional public agricultural brands. The specific indicators are defined as follows:

Let V denote variety renewal, a binary variable that takes the value of 1 if variety renewal occurs and 0 otherwise. Let S denote the degree of production standardization, which is affected by variety renewal. The parameter φ represents the horticultural-crop characteristic coefficient, capturing the fact that, compared with field crops, horticultural crops may experience a greater short-term decline in production standardization after variety renewal. Let R denote returns to scale, which are specified as a function of production standardization. Let C represent production costs, which are influenced by returns to scale. Let Q denote grape yield, which is affected by production costs and brand value, and let P denote the unit price of grapes, which is affected by grape variety renewal and brand value. TR denotes farmers’ total revenue from grape production, while π denotes net farm income. To capture the spillover effects of branding, this study further incorporates the value of the regional public brand, denoted by B, and the regional public brand spillover coefficient, denoted by θ. In addition, α represents the quality and scarcity premium associated with new varieties.

First, changes in production standardization before and after variety renewal are specified as follows. Before variety renewal occurs, that is, when V = 0, the degree of production standardization is denoted by S_0_, such that S(V)=S_0_. After variety renewal occurs, that is, when V = 1, the degree of production standardization is denoted by S_1_, such that S(V)=S_1_=φS_0_, where φ∈[0,1]. This specification reflects the assumption that grape variety renewal may reduce production standardization in the short term because farmers need time to adapt to new cultivation techniques and management requirements. Therefore, S_0_ > S_1_.

Second, examine the effect of production standardization on returns to scale. Generally, returns to scale increase as the degree of production standardization increases (Argente et al., 2025), Let R = f(S), then, *dR/dS* > 0. When S_0_ > S_1_, we have R_0_ > R_1_.

Third, we derive the functional form of production costs with respect to returns to scale, Let C=f(R). As returns to scale change, production costs also change (Panagariya, 1981). Generally, the higher the returns to scale, the lower the production costs; conversely, the lower the returns to scale, the higher the production costs. Therefore, *dC/dR* < 0. From this, we can see that when R_0_ > R_1_, C_0_ < C_1_.

Fourth, examine the effects of production costs and brand value on output. Let Q = f(C, B). When costs rise, profits are squeezed, forcing prices to increase and leading to a decline in market share (Boulding and Staelin, 1993). At the same time, brand value helps mitigate the decline in output, but its effect is far less significant than the dampening effect of rising costs on output. Therefore, Q_0_ > Q_1_.

Fifth, the impact of variety renewal and brand spillover on the unit price of the product. Let the unit price of grapes before the variety renewal be P_0_, and after the variety renewal be P_1_. On the one hand, the quality and scarcity of the new variety drive prices upward; on the other hand, brand value generates positive externalities, and this value spillover manifests as a further increase in price. Therefore, we have P_1_ = P_0_(1+αV)(1+θB). Since α > 0 and θ > 0, P_1_ > P_0_.

Sixth, Price elasticity of demand and total revenue. Generally, demand for agricultural products is inelastic, and this holds true for horticultural crops such as grapes. That is, E_d_ < 1, meaning that the increase in price is far greater than the decrease in quantity. Farmers’ total revenue is TR = P*Q. The increase in P_1_ relative to P_0_ following the variety change is significantly greater than the decrease in Q_1_ relative to Q_0_. Let TR_1_ denote the farmers’ total revenue after the variety renewal and TR_0_ denote the farmers’ total revenue before the variety renewal. Then, TR_1_ > TR_0_.

From an economic perspective, and based on the theory of returns to scale and the characteristics of horticultural crop production, grapes, as a perennial horticultural crop, rely heavily on stable and standardized management practices as well as well-established mechanisms for achieving returns to scale. Variety renewal may disrupt production routines, management standards, and labor-division systems that have been developed over a long period. In the short term, this disruption may reduce the degree of production standardization, weaken returns to scale at different stages of production, and increase unit production costs. Constrained by short-term input adjustments and the growth cycle of newly planted vines, farmers may experience a temporary reduction in effective production scale and yield. Moreover, because the adjustment costs associated with horticultural crop variety renewal are generally higher than those for field crops, the short-term suppression of production capability may be more pronounced. Therefore, we propose:

Hypothesis 1: All else being equal, horticultural crop variety renewal is likely to reduce crop yield in the short term.

Based on cost–benefit theory and the spillover effects of regional public brands, variety renewal may increase product prices through several mechanisms. On the one hand, new varieties often possess improved appearance, taste, storability, or stress tolerance, and these quality advantages may increase consumers’ willingness to pay, thereby generating a variety-quality premium. On the other hand, the unified replacement of varieties across a region enhances the consistency and distinctiveness of products within that region, strengthening the market recognition and reputation of the regional public brand; the spillover effects of the regional public brand will further amplify the product’s premium potential. At the same time, the short-term contraction in market supply caused by variety renewal may create relative scarcity. In addition, rising unit production costs may be partially passed through to market prices. Together, these mechanisms may increase the unit price of horticultural products. Based on this, we propose:

Hypothesis 2: All else being equal, horticultural crop variety renewal is expected to increase the market unit price of agricultural products.

Based on the theory of price elasticity of demand and the income-composition logic of agricultural production, horticultural crops, as fresh agricultural products, exhibit strong inelasticity in consumer demand, their price elasticity of demand is significantly less than 1, meaning that price changes have a relatively weak impact on the quantity demanded. Under this condition, the price premium generated by variety renewal may offset the revenue loss caused by the short-term decline in marketable yield. Since farmers’ total income from grape production is determined by unit price and marketable yield, an increase in unit price may lead to higher total income if the proportional increase in price exceeds the proportional decline in yield. In addition, the spillover effects of regional public brands may further enhance the price premium associated with variety renewal, thereby strengthening its positive effect on farmers’ total income. Therefore, we propose:

Hypothesis 3: All else being equal, horticultural crop variety renewal is expected to increase farmers’ total income in the short term.

**Figure 1:**
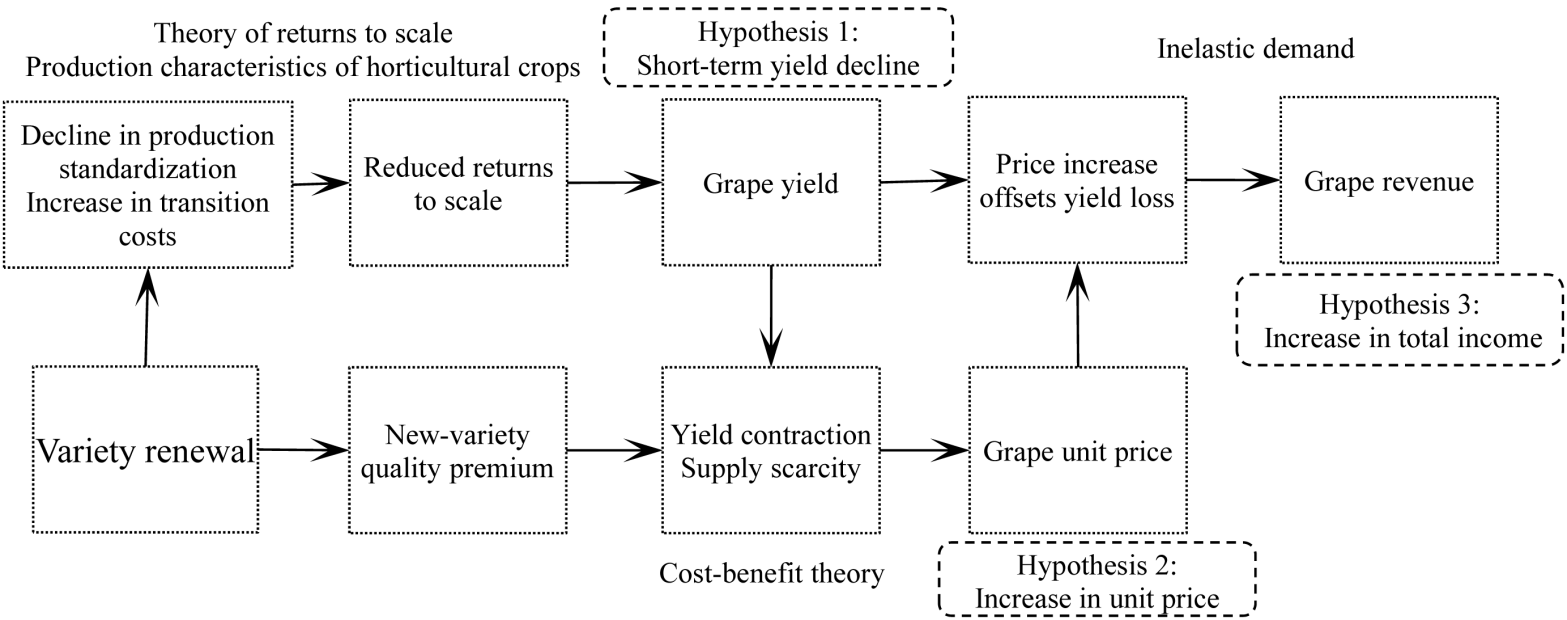
Theoretical framework.

The theoretical framework is presented in **Figure 1**.

### 2.2 Data sources

The data in this article is derived from a sample survey conducted by the Agricultural Brand Research Center at Shenyang Agricultural University between July and August 2023 among grape growers in the major grape−growing regions of Liaoning Province. Liaoning Province has a long history of grape cultivation; its grape−growing area and total output rank second in China, making it highly representative and capable of accurately reflecting the actual growth patterns of the crop under study. This paper focuses solely on data collected by the research team during their fieldwork in Gaizhou City, Liaoning Province, for the following reasons: First, Gaizhou City has a large grape−growing area and a highly mature industry. The primary variety produced is Kyoho grapes; grapes from Gaizhou are known as “Southern Liaoning Grapes,” which have a wide sales reach and strong brand recognition (Yang et al., 2026); Second, the grape industry in Gaizhou has undergone significant renewal; influenced by market trends, many farmers have switched to growing Shine Muscat grapes, which had already reached a substantial scale by 2023. Therefore, the grape industry in Gaizhou provides a highly suitable data sample for this study, facilitating the analysis of the specific impact of variety renewal on the brand effect of agricultural products.

To ensure data quality, the research team conducted thorough preliminary analysis and developed a meticulously planned survey scheme. In Gaizhou City, survey sites and sample households were selected using a combination of stratified purposive sampling and random sampling. A research team consisting of 28 doctoral and master’s students conducted one-on-one questionnaire surveys with grape-growing households. Based on the distribution of major grape-producing areas and fieldwork feasibility, six townships or subdistricts in Gaizhou City were selected as survey sites: Dongcheng Subdistrict, Jiuzhai Town, Ertai Township, Tuandian Town, Shagang Town, and Chentun Town. The sample size for each township or subdistrict was allocated according to its grape planting area, with sampling proportions of 5% for Dongcheng Subdistrict, 24% for Jiuzhai Town, 23% for Ertai Township, 22% for Tuandian Town, 2% for Shagang Town, and 24% for Chentun Town. After arriving at each survey site, the research team randomly selected farmers from lists provided by local agricultural officials. The selected farmers had been continuously engaged in grape cultivation for many years, independently managed their own vineyards, and cultivated Kyoho or Shine Muscat grapes. Farmers who had renewed varieties in 2022 but had no harvest in that year were excluded from the sample. A total of 487 data points were collected. After excluding samples with missing key information, 444 valid samples remained, resulting in a sample validity rate of 91.7%.

Grape growers in Gaizhou City are generally older and have been engaged in grape production for many years. Descriptive statistics show that the interviewed grape growers had an average age of 51 years, approximately 19 years of grape cultivation experience, and an average planting area of about 7 mu. These characteristics are broadly consistent with the typical profile of fresh table grape growers in northern China, suggesting that the sample is reasonably representative. It should be noted that the transition from Kyoho to Shine Muscat among Gaizhou grape growers was relatively concentrated over time. Driven by large-scale growers and market trends, most surveyed growers adopted Shine Muscat after 2020. The vines did not bear fruit in the year of renewal but began producing fruit in the following year. Since the field survey focused primarily on grape production in 2022, growers who renewed their varieties by adopting Shine Muscat in 2022 were excluded from the analysis.

### 2.3 Variable selection and description

Dependent variable. The concept of regional public brands of agricultural products primarily stems from relevant documents issued by China’s Ministry of Agriculture and Rural Affairs (Liu and Wang, 2023). These brands draw upon the natural ecological environment and historical and cultural factors of specific regions, serving as symbolic brand identifiers for industrial clusters within those regions (Fromhold-Eisebith and Eisebith, 2005). Furthermore, such brands are jointly used by multiple agricultural production and business entities (Björner and Aronsson, 2022), and often adopts a naming format of “place of origin + product name” (Kavaratzis and Hatch, 2013). Considering that successful regional public brands of agricultural products can sustainably promote agricultural development and ultimately effectively increase farmers’ income levels (Yin et al., 2024; Castillo-Diaz et al., 2023), this study uses three indicators—grape yield, revenue, and unit price—to measure the upgrading and development of regional public brands of agricultural products. Grape yield is represented by the annual total grape production (in 5 metric tons) of the surveyed households; grape revenue is represented by the annual gross income (in CNY 10000) from grape cultivation; and grape unit price is calculated as the ratio of revenue to sales volume.

Core independent variable. This paper primarily examines the impact of changing grape varieties on brand effects. Variety renewal serves as the core explanatory variable in this study. Regarding rice variety renewal, variety renewal refers to a change in the variety or category of crops being cultivated, typically involving the replacement of older varieties with newer ones (Atlin et al., 2017; Song et al., 2015). Based on the actual conditions of Gaizhou grapes, this study categorizes variety renewal into two levels: First, variety renewal, which reflects the actual situation where growers uproot the original Kyoho grape variety and switch to Shine Muscat grapes. This is a binary variable (0–1), where 0 indicates no replacement behavior and 1 indicates replacement behavior; Second, the degree of variety renewal, which is a multi−category variable: 1 indicates no replanting, 2 indicates a complete switch to Shine Muscat grapes, and 3 indicates partial replanting (where a portion is replanted with Shine Muscat grapes while the remainder continues to be planted with Kyoho grapes).

Other variables.

① Instrumental variables. To address the endogeneity issue caused by samples’ self-selection, and following previous research (Gitter et al., 2012), this study uses the average value of variety renewal choices among other farmers in the same village, excluding the respondent, as an instrumental variable for the respondent’s variety renewal choice, denoted as IV_variety_renewal. On the one hand, whether other farmers in the same village choose variety renewal may influence the respondent’s own choice through peer effects and information spillovers, indicating a strong correlation between the instrumental variable and the endogenous variable. On the other hand, the average value of the variety renewal choice variable among other farmers in the same village is unlikely to directly affect the respondent’s grape yield, income, or unit price, except through the respondent’s own variety renewal choice. Therefore, this instrumental variable satisfies the relevance condition and the exclusion restriction.

② Mechanism variables. Variety renewal may influence farmers’ production input decisions, improve product quality, and enhance brand premiums, thereby affecting both grape yield and farmers’ income. In agricultural production, labor input, facility upgrading, and technology adoption are important components of farmers’ production input decisions. However, these factors are difficult to observe directly, and reliable data on them are often difficult to obtain. By contrast, the application amounts of chemical fertilizers and farmyard manure are tangible and observable production inputs, and data on these inputs are more readily available. Therefore, this study uses the application rates of chemical fertilizers and farmyard manure to capture changes in production inputs among grape growers and to examine whether variety renewal affects yield and income by altering production input decisions. Specifically, the chemical fertilizer application rate is defined as the per-mu amount of chemical fertilizers used by the surveyed households during the 2022 grape production cycle, including nitrogen–phosphorus–potassium compound fertilizers, foliar fertilizers, and water-soluble fertilizers. It is measured in jin per mu and treated as a continuous variable. The farmyard manure application rate is defined as the per-mu amount of manure-based organic fertilizers, such as sheep manure and pig manure, used by the surveyed households during the 2022 grape production cycle. It is also measured in jin per mu and treated as a continuous variable.

③ Moderator variables. This study further examines whether the effects of variety renewal on yield and income differ across five moderating dimensions: farmer training, information access capability, online sales capability, social learning, and social capital networks. First, farmer training refers to the number of times grape growers participated in grape production- and management-related training activities organized by government departments, cooperatives, enterprises, or other organizations within a given year. It is measured using the survey item “the number of times growers attended grape−related training sessions in a year” and is treated as a continuous variable. Second, information access capability reflects the extent to which consumers can obtain information (Yuan and Xiao, 2021). In the daily lives of grape growers, the adequacy of their information access is closely related to the frequency with which they obtain relevant information. Therefore, this study measures information access capability using the survey item “the number of times growers used internet tools such as mobile phones to access grape−related information.” This variable is treated as continuous, with higher values indicating stronger information access capability. Third, farmers’ participation in online sales mainly takes two forms: directly selling agricultural products through online platforms, and supplying products to cooperatives or other organizations that operate online sales platforms (Cheng et al., 2018). In Gaizhou, grape growers mainly participate in online sales through the former channel. Therefore, this study measures online sales participation using the survey item “Have you ever sold your grapes via WeChat, Weibo, QQ, or similar platforms?” This is a binary variable, where 1 indicates participation in online sales and 0 otherwise. Fourth, social learning refers to growers’ active behavior in acquiring information and knowledge related to grape production and marketing. It involves a dynamic process of searching for, absorbing, and applying new knowledge and innovations (Barham et al., 2015) and includes the important dimension of mass media learning. This study measures social learning using the survey item “The number of times, over the past year, growers have proactively learned about grape production and sales through mobile phones, computers, books, or other resources.” This variable is treated as continuous, with higher values indicating a higher level of social learning. Fifth, the level of social capital networks includes whether respondents have relatives or friends working in government agencies or schools (Zhang et al., 2015). If such relatives or friends exist, farmers can obtain cutting-edge information on market dynamics, which facilitates making sound production and business decisions. The study measured the level of social capital networks among grape growers using the question: “The number of the respondent’s relatives and friends who work in government.” This is a continuous variable, with higher values indicating a stronger level of social capital networks.

④ Control variables. Based on the literature (Shah et al., 2020; Abrha, 2015; Jakperik and Oduro, 2013; Javed et al., 2003), this study sequentially included variables such as cultivated area, input costs, greenhouse use, age, health status, education, years of farming, and township to control for these factors in the regression analysis. All data were derived from field survey data collected in Gaizhou City in 2023, the statistical scope covers the production and business operations of grape growers in Gaizhou in 2022. Descriptive statistics for the variables are presented in **Table 1**.

**Table 1:** Descriptive statistics.

Variable type	Variable	Variable description	Mean	Standard deviation	Min	Max
Dpendent variable	Grape yield	annual production	3.007	1.966	0.35	17
	Grape revenue	annual income	11.388	8.779	0.8	50
	Grape uint price	CNY	4.081	3.081	0.36	30
Independent variables	Variety renewal	0=no replacement 1=repalcement	0.466	0.499	0	1
	Degree of variety renewal	1=no new varieties 2=all replacement 3=partial replacement	1.759	0.877	1	3
Instrumental variables	Iv_variety renewal	the average value of variety renewal choices among other farmers in the same village	0.385	0.193	0.09	0.89
Mechanism variables	Chemical fertilizer	amount of chemical fertilizer used per mu	485.905	492.702	0	3900
	Farmyard manure	amount of farmyard manure used per mu	2144.461	1844.108	0	8571
Moderator variables	Training	number of training sessions per year	1.864	1.797	0	10
	Information access capability	frequency of use of IT tools	2.327	4.482	0	30
	Online sales capability	should we use tools like WeChat to sell grapes?	0.087	0.282	0	1
	Social learning	number of active learning sessions	1.717	6.762	0	60
	Social capital networks	number of relatives and friends working in government	0.891	2.042	0	15
Control variables	Planted area	Area under grape cultivation	7.448	5.153	1	40
	Input cost	Annual Farming Expenses	3.044	3.614	0.2	35
	Greenhouse	Is there a greenhouse?	0.599	0.490	0	1
	Age	years	51.560	8.535	28	72
	Health status	Level of health	2.832	0.426	1	3
	Education	Length of education	2.000	0.689	1	5
	Growing period	Number of years of continuous grape cultivation	19.470	8.968	2	50
	Town	Dongcheng, Jiuzhai, Ertai, Tuandian, Shagang, Chentun	6.982	5.287	2	17

## 3 Empirical analysis

### 3.1 Regression analysis

The study used three indicators—grape yield, revenue, and unit price—to reflect the brand effect, and performed multiple linear regression analyses on these three indicators against variety renewal. Based on cost-benefit theory and the literature (Boulding and Staelin, 1993; Shah et al., 2020; Abrha, 2015; Jakperik and Oduro, 2013; Javed et al., 2003), control variables were included, such as planted area, costs, greenhouses, training, education, online sales capability, information access capability, social capital networks, and township. The multiple linear regression model was set up as follows,

(1) $$y_{1}=\alpha_{1}+\;\beta_{1}x+\theta_{1}z_{1}+\epsilon_{1}$$

(2) $$y_{2,3}=\alpha_{2,3}+\;\beta_{2,3}x+\theta_{2,3}z_{2}+\epsilon_{2,3}$$

In this model as shown in Equations (1, 2), y_1_ represents grape yield, y_2_ and y_3_ represent revenue and unit price respectively, and x represents variety renewal. z_1_ represents the control variables affecting grape yield, generally, a larger planting area is associated with higher grape production, while input costs reflect the intensity of production inputs and are closely related to grape yield. Farmers’ human capital also affects the quality of field management, which is particularly important for grape production and may significantly influence grape yield. In addition, given the actual production conditions in Gaizhou City, greenhouse use can help reduce disaster risks and stabilize grape production. Therefore, this study includes planted area, input costs, training, education, greenhouse, and township as control variables in the grape yield model. z_2_ represents the control variables affecting grape revenue and unit price, variables that affect grape yield may also affect grape revenue and are therefore included as control variables. In addition, farmers’ income is related not only to traditional cost expenditure but also to their ability to apply digital technologies. Therefore, this study includes variables that measure farmers’ online sales capability and information access capability as control variables. Previous studies have shown that social capital networks affects farmers’ income. For grape growers in Gaizhou City, non-agricultural income is also an important substitute for agricultural income. Accordingly, this study includes planted area, input costs, greenhouse, training, education, online sales capability, information access capability, social capital networks, non-agricultural income, and township as control variables in the grape revenue model. Due to data limitations and for ease of comparison, the same set of variables is also used as control variables in the unit price model. α_1_, α_2_ and α_3_ represent the constant terms, while ϵ_1_, ϵ_2_ and ϵ_3_ represent the random disturbance terms. To ensure that the model specifications were appropriate, this study conducted Ramsey RESET tests for each of the three regression models. The p-values were 0.107, 0.450, and 0.468, respectively. All tests passed the null hypothesis, indicating no evidence of model misspecification and suggesting that the linear specification was appropriate. The regression results are shown in **Table 2**. The study focuses on the coefficients β_1_, β_2_ and β_3_. As shown in the table, the impact of variety renewal on the above three indicators all passed the significance levels of 5%, 5%, and 1%. Variety renewal resulted in a significant decrease in grape yield, a significant increase in grape revenue, and a significant increase in grape unit price.

**Table 2:** The brand effect of variety renewal.

Variables	Grape yield (1)	Grape revenue (2)	Grape unit price (3)
Variety renawal	-0.384** (0.169)	1.573** (0.751)	0.302*** (0.060)
Planted area	0.294*** (0.031)	0.911*** (0.090)	-0.008 (0.005)
Input cost	0.001*** (0.000)	-0.003** (0.001)	-0.000*** (0.000)
Greenhouse	-0.337* (0.191)	1.574** (0.668)	0.236*** (0.055)
Training	0.002*** (0.000)	0.001 (0.001)	-0.001*** (0.000)
Education	0.037 (0.102)	0.611 (0.487)	0.030 (0.036)
Online sales capability	–	0.103 (0.242)	0.028 (0.021)
Information access capability	–	1.955* (1.176)	0.168** (0.084)
Social capital networks	–	1.332** (0.564)	0.079 (0.048)
Non-agricultural income	–	-0.037 (0.091)	0.001 (0.007)
Town	Control	Control	Control
Constant	1.009** (0.418)	4.604** (1.898)	1.341*** (0.124)
Obs	443	444	443
R^2^	0.474	0.480	0.258

The figures in parentheses represent robust standard errors; ***, ** and * denote significance levels of 1%, 5% and 10%, respectively.

From the economic interpretation of the regression results, variety renewal is associated with a reduction in annual grape yield of approximately 3,800 jin, while farmers’ total annual income increases by about 16,000 yuan. The unit price of Shine Muscat is substantially higher than that of Kyoho, with the purchase price increasing by approximately 1.57 yuan per jin. After controlling for planting scale, facilities and equipment, and farmer characteristics, farmers who adopted variety renewal achieved higher total income than those who continued to cultivate traditional Kyoho grapes, although their yield declined to some extent. This finding is consistent with the economic logic of grape growers pursuing higher income through variety renewal. The variety renewal process in Gaizhou City is still at an early stage, and the transition period has been relatively short. Because the yield of newly adopted varieties has not yet reached its mature production level, farmers may experience a temporary decline in yield during the initial stage of renewal. This reflects the short-term production characteristics of fresh table grape variety renewal in Gaizhou, where the productivity potential of the new variety has not yet been fully realized. At the same time, the unit price of Shine Muscat is substantially higher than that of traditional Kyoho, which is a key reason for the increase in farmers’ total income. From the perspective of crop production and market dynamics, Shine Muscat currently benefits from a short-term price premium generated by supply scarcity and quality differentiation. Although this premium increases farmers’ total revenue, production capability has not yet reached the normal level for mature vines, and supply fluctuations may affect the stability of the regional brand reputation. In the long term, as Shine Muscat enters its full-bearing stage and market supply expands, its current price premium may gradually weaken, which may further affect the stability of brand value.

### 3.2 Robustness tests

To further test the robustness of the baseline regression results, this study employs two robustness checks. First, the dependent variables are log-transformed and then re-estimated. The results are shown in columns 1–3 of **Table 3**. The effects of variety renewal on grape yield, grape revenue, and unit price are significant at the 1%, 5%, and 1% levels, respectively. In terms of coefficient signs, variety renewal leads to a decrease in grape yield while increasing grape revenue and unit price, which is consistent with the baseline regression results. Second, this study applies 5% two-tailed winsorization to the dependent variables. The results are presented in columns 4–6. Similar to the previous robustness check, the effects of variety renewal on grape yield, grape revenue, and unit price are significant at the 5%, 10%, and 1% levels, respectively. The coefficient signs also remain unchanged, indicating that variety renewal reduces grape yield but increases grape revenue and unit price. Taken together, the results of both robustness checks remain substantively unchanged and are consistent with the baseline regression results, indicating that the empirical conclusions of this study are robust.

**Table 3:** Results of the robustness tests.

Variables	Log-transformed dependent variables			Winsorized dependent variables		
	Grape yield (1)	Grape revenue (2)	Grape unit price (3)	Grape yield (4)	Grape revenue (5)	Grape unit price (6)
Variety renawal	-0.191*** (0.064)	0.154** (0.072)	1.670*** (0.316)	-0.328** (0.154)	1.286* (0.665)	1.212*** (0.212)
Planted area	0.097*** (0.009)	0.094*** (0.009)	-0.074*** (0.026)	0.255*** (0.019)	0.810*** (0.076)	-0.049** (0.019)
Input cost	0.000*** (0.000)	-0.000*** (0.000)	-0.001*** (0.000)	0.000*** (0.000)	-0.002*** (0.000)	-0.001*** (0.000)
Greenhouse	-0.053 (0.062)	0.105* (0.063)	0.991*** (0.246)	-0.207 (0.158)	1.539** (0.616)	0.885*** (0.193)
Training	-0.001*** (0.000)	0.000 (0.000)	0.006*** (0.000)	-0.001*** (0.000)	-0.000 (0.001)	0.005*** (0.000)
Education	0.030 (0.038)	0.042 (0.046)	0.094 (0.178)	0.074 (0.087)	0.520 (0.412)	0.108 (0.132)
Online sales capability		0.019 (0.028)	-0.181** (0.089)		-0.066 (0.212)	-0.157** (0.066)
Information access capability		0.236*** (0.090)	1.285 (0.782)		2.072** (0.961)	0.481* (0.272)
Social capital networks		0.058 (0.061)	0.467** (0.218)		1.102** (0.497)	0.287* (0.156)
Non-agricultural income		-0.014 (0.010)	-0.039 (0.039)		-0.021 (0.080)	0.004 (0.029)
Town	Control	Control	Control	Control	Control	Control
Constant	0.015 (0.155)	1.167*** (0.108)	4.508*** (0.722)	0.790** (0.340)	4.582*** (1.640)	4.415*** (0.488)
Obs	443	444	443	443	444	443
R2	0.432	0.410	0.232	0.488	0.500	0.312

The figures in parentheses represent robust standard errors; ***, ** and * denote significance levels of 1%, 5% and 10%, respectively.

### 3.3 Endogeneity tests

To test the robustness of the baseline regression results and address the endogeneity issuesarising from sample self-selection, this study employs an instrumental variable approach to mitigateestimation biases potentially caused by endogeneity. The instrumental variable is Iv_variety renewal, which is calculated as the average value of variety renewal choices among other farmers in the same village. To further test the suitability of the instrumental variable, the study conducted a weak instrumental variable test for Iv_variety renewal to examine whether the instrumental variable coefficients in the first stage of the two-stage estimation were zero. Following the conventional rule of thumb, a first-stage F-statistic greater than 10 indicates that the hypothesis that “weak instrumental variables exist” can be rejected. Because this study estimates three separate two-stage least squares models for grape yield, grape revenue, and unit price, weak-instrument tests are reported for each model. The corresponding first-stage F-statistics are 10.94, 10.86, and 10.69, respectively (see Appendix Table 1), all exceeding the conventional threshold of 10. These results suggest that Iv_variety renewal is sufficiently correlated with variety renewal and can serve as a relevant instrumental variable in the first-stage regressions.

This study employs a two-stage least squares method for regression estimation. The results of the first-stage regressions are shown in columns 1, 4, 7 of **Table 4**. The estimated values of Iv_variety renewal on variety renewal are incorporated into the next stage of the regression process to examine its effects on grape yield, grape revenue, and grape unit price, respectively. The results are shown in columns 2 5 8 respectively. As shown in the table, after controlling for endogeneity biases potentially arising from sample self-selection, variety renewal has a significant positive effect on grape revenue and grape unit price, with significance levels of 5% and 1%, respectively. Although it does not significantly affect grape yield, the coefficient is negative, which is consistent with the conclusions of the previous section. This provides some validation of the robustness of the baseline regression results.

**Table 4:** Results of the endogeneity test.

Variables	Grape yield			Grape revenue			Grape unit price		
	First stage (1)	2sls (2)	Liml (3)	First stage (4)	2sls (5)	Liml (6)	First stage (7)	2sls (8)	Liml (9)
Variety renewal		-0.217 (1.096)	-0.217 (0.968)		12.207** (5.111)	12.207** (5.007)		6.772*** (2.238)	6.772*** (2.214)
Iv_variety renewal	0.379*** (0.114)			0.375*** (0.114)			0.373*** (0.114)		
Planted area	0.026*** (0.004)	0.279*** (0.038)	0.279*** (0.031)	0.025*** (0.004)	0.704*** (0.162)	0.704*** (0.159)	0.025*** (0.004)	-0.187*** (0.068)	-0.187*** (0.069)
Input cost	-0.000*** (0.000)	0.000** (0.000)	0.000 (0.001)	-0.000* (0.000)	-0.001 (0.001)	-0.001 (0.005)	-0.000* (0.000)	-0.000 (0.000)	-0.000 (0.002)
Greenhouse	0.333*** (0.045)	-0.512 (0.457)	-0.512 (0.375)	0.322*** (0.045)	-2.676 (2.021)	-2.676 (1.886)	0.325*** (0.045)	-0.970 (0.886)	-0.970 (0.838)
Training	0.001*** (0.000)	-0.002** (-0.000)	-0.001 (0.001)	0.000*** (0.000)	-0.007** (0.003)	-0.007 (0.009)	0.000*** (0.000)	0.003* (0.001)	0.003 (0.004)
Education	0.015 (0.030)	0.046 (0.103)	0.046 (0.104)	0.007 (0.032)	0.510 (0.580)	0.510 (0.544)	0.007 (0.032)	0.050 (0.226)	0.050 (0.239)
Health status				0.048 (0.037)	-0.005 (0.680)	-0.005 (0.783)	0.049 (0.037)	-0.007 (0.269)	-0.007 (0.345)
Online sales capability				-0.003 (0.015)	-0.086 (0.261)	-0.086 (0.303)	-0.003 (0.015)	-0.180 (0.110)	-0.180 (0.133)
Information access capability				0.081 (0.074)	1.353 (1.430)	1.353 (1.286)	0.079 (0.074)	1.004 (0.847)	1.004* (0.564)
Social capital networks				0.024 (0.040)	1.064 (0.709)	1.064 (0.728)	0.026 (0.040)	0.331 (0.300)	0.331 (0.320)
Non-agricultural income				-0.000 (0.006)	-0.005 (0.120)	-0.005 (0.126)	-0.000 (0.006)	-0.028 (0.053)	-0.028 (0.055)
Constant	-0.135** (0.066)	1.028*** (0.250)	1.028*** (0.217)	-0.268** (0.122)	0.391 (1.983)	0.391 (2.347)	-0.268** (0.122)	2.609*** (0.837)	2.609** (1.031)
Obs	443	443	443	444	444	444	443	443	443
R2	0.284	0.447	0.447	0.289	0.199	0.199	0.290	0.220	0.220

The figures in parentheses represent robust standard errors; ***, ** and * denote significance levels of 1%, 5% and 10%, respectively.

Subsequently, to further test the robustness of the baseline regression results and assess whether the findings are sensitive to potential weak-instrument concerns, this study re-estimates the model using the Limited Information Maximum Likelihood (LIML) method, which generally has better finite-sample properties than 2SLS under weak identification. The LIML results (columns 3, 6, 9) show that variety renewal has a positive effect on grape revenue and grape unit price, with the coefficients being statistically significant at the 5% and 1% levels, respectively. The effect on grape yield is not statistically significant, which is consistent with the 2SLS results reported above. The direction and statistical significance of the main results remain broadly consistent, suggesting that the core conclusions are robust.

### 3.4 Quantile regression analysis

In reality, the impact of variety renewal on production varies among farmers of different levels. To address this, this paper employs quantile regression to examine the differences in the impact of variety renewal on the brand effect across different farmer types. At the same time, farmers adopt different approaches when updating grape varieties. Some farmers choose to completely remove Kyoho grapes and replant them with Shine Muscat grapes, while others remove only a portion of the Kyoho grapes, replanting the rest with Shine Muscat grapes and retaining a portion of the Kyoho grapes. The extent of variety renewal has cumulative impacts on production outcomes among farmers at different levels.

This study employs quantile regression at the 10th, 25th, 50th, 75th, and 90th percentiles to examine the heterogeneous effects of variety renewal on grape yield. The results are reported in **Table 5**. To determine whether the quantile regression results differed significantly across different quantiles, this study conducted a Wald test for joint significance on the quantile regression process. The results show an F-value of 2.59 and a p-value of 0.009, indicating that the effect of variety renewal on grape yield differed significantly across the aforementioned quantile points and that quantile regression was appropriate. For farmers adopting full renewal, grape yield decreases significantly among farmers with medium or lower yield levels, corresponding to the 10th, 25th, and 50th percentiles, the magnitude of the yield decline also decreases accordingly. For farmers with higher yield levels adopting full renewal, the estimated effect on yield is not statistically significant. For farmers adopting partial renewal, grape yield does not change significantly among farmers with the lowest and highest yield levels, corresponding to the 10th and 90th percentiles, respectively. However, for farmers with medium and near-medium yield levels, corresponding to the 25th, 50th, and 75th percentiles, partial renewal is associated with a significant decline in yield, and the magnitude of the decline also increases accordingly. These findings suggest that, under full renewal, the production capacity of farmers with medium or lower yield levels has not yet been fully realized, possibly because these farmers have weaker capability to absorb transition risks. Under partial renewal, farmers with medium yield levels appear to face fragmented adjustment problems arising from mixed cultivation of old and new varieties and inconsistent management practices, indicating limitations in scale adaptation during fruit-tree variety renewal. From the perspective of cost–benefit heterogeneity, farmers with different yield levels exhibit substantial differences in returns during the variety renewal process. Farmers with low yield levels are typically characterized by low-input and fragmented production. Although their total transition costs are relatively low, they face a higher risk of yield loss under full renewal, whereas partial renewal appears to be a more stable strategy. Farmers with medium yield levels face the dual challenge of managing both old and new varieties, while the returns to technical investment may remain limited. As a result, they may incur relatively high unit production costs and face pressure from both yield decline and rising costs under either full or partial renewal. Farmers with high yield levels, by contrast, can rely on centralized procurement and standardized management to spread costs and reduce unit production costs. Full renewal enables them to capture quality premiums more effectively, while partial renewal allows them to balance stable production with relatively high returns, suggesting stronger resilience to market and production risks.

**Table 5:** Quantile results on the effect of variety renewal on grape yield.

Variables	Grape yield				
	10th percentile (1)	25th percentile (2)	50th percentile (3)	75th percentile (4)	90th percentile (5)
Complete variety renewal	-0.701*** (0.220)	-0.533* (0.291)	-0.432** (0.178)	-0.157 (0.202)	0.450 (0.387)
Partial variety renewal	-0.028 (0.287)	-0.499** (0.232)	-0.528*** (0.148)	-0.627*** (0.173)	-0.366 (0.389)
Planted area	0.190*** (0.028)	0.249*** (0.024)	0.322*** (0.027)	0.421*** (0.050)	0.455*** (0.055)
Input cost	0.002 (0.029)	0.001 (0.032)	0.001 (0.021)	0.000 (0.015)	-0.001 (0.008)
Greenhouse	-0.154 (0.296)	-0.005 (0.159)	0.002 (0.159)	-0.154 (0.164)	-0.500* (0.292)
Training	0.002 (0.041)	0.002 (0.050)	0.002 (0.056)	0.002 (0.037)	0.002 (0.046)
Town	Control	Control	Control	Control	Control
Constant	0.436 (0.340)	0.254 (0.329)	0.267 (0.426)	0.315 (0.311)	0.779 (1.160)
Obs	443	443	443	443	443
R^2^	0.205	0.281	0.341	0.381	0.377

The figures in parentheses represent robust standard errors; ***, ** and * denote significance levels of 1%, 5% and 10%, respectively.

This study employs quantile regression at the 10th, 25th, 50th, 75th, and 90th percentiles to examine the heterogeneous effects of variety renewal on grape revenue. The results are reported in **Table 6**. To determine whether there were significant differences in the quantile regression results across different quantiles, this study conducted a Wald test for joint significance of the quantile regression process. The results showed an F-value of 8.24 and a p-value of 0.000, indicating that the effect of variety renewal on grape revenue differed significantly at the aforementioned quantile points, and that quantile regression was an appropriate method. For farmers adopting full renewal, grape revenue increases significantly among farmers with medium or higher revenue levels, corresponding to the 50th, 75th, and 90th percentiles, and the magnitude of the revenue increase rises as the revenue level increases. For farmers with lower revenue levels adopting full renewal, the estimated effect on grape revenue is not statistically significant. For farmers adopting partial renewal, grape revenue decreases significantly among farmers with medium and relatively higher revenue levels, corresponding to the 50th and 75th percentiles. These results indicate that, under full renewal, farmers with medium or higher revenue levels are better able to capture the price premium generated by new varieties. By contrast, for farmers with lower revenue levels, the variety premium may be insufficient to offset yield losses and adjustment costs, resulting in no significant increase in income. Under partial renewal, farmers with medium and relatively higher revenue levels may face higher unit costs due to the simultaneous management of old and new varieties. In this case, the price premium generated by the renewed varieties may be insufficient to compensate for the additional costs associated with mixed cultivation. From the perspective of cost–benefit heterogeneity, the operational performance of farmers differs substantially across revenue-level groups and renewal patterns. For farmers with low revenue levels adopting full renewal, yield remains relatively low, but the price premium of the new variety may offset part of the yield loss, thereby keeping overall income relatively stable. Farmers with medium revenue levels face higher opportunity costs associated with mixed cultivation. Those adopting full renewal may achieve higher income by relying on relatively stable production capability and the price premium of new varieties, whereas those adopting partial renewal may experience both reduced production capability and lower income. Farmers with high revenue levels adopting full renewal are better positioned to capture the quality premium of new varieties and generate higher income. These findings suggest that grape variety renewal is not merely a simple substitution of one variety for another; rather, its income-enhancing effect depends on clear scale thresholds and the suitability of the renewal pattern.

**Table 6:** Quantile results on the impact of variety renewal on grape revenue.

Variables	Grape revenue				
	10th percentile (1)	25th percentile (2)	50th percentile (3)	75th percentile (4)	90th percentile (5)
Complete variety renewal	1.692 (1.373)	1.863 (1.202)	4.589** (2.028)	8.356** (3.827)	14.349*** (5.090)
Partial variety renewal	0.120 (0.580)	-1.098 (0.865)	-1.350** (0.575)	-1.786** (0.834)	0.478 (1.562)
Planted area	0.688*** (0.075)	0.891*** (0.117)	1.141*** (0.159)	1.329*** (0.185)	1.508*** (0.242)
Input cost	-0.001 (0.167)	-0.002 (0.303)	-0.003 (0.423)	-0.005 (0.377)	-0.006 (0.221)
Greenhouse	-0.235 (0.712)	0.645 (0.731)	0.715 (0.585)	1.580 (1.045)	4.305*** (1.407)
Training	-0.001 (0.154)	0.000 (0.119)	0.002 (0.104)	0.005 (0.155)	0.014 (0.364)
Online sales capability	0.223 (0.227)	-0.001 (0.218)	0.033 (0.177)	0.054 (0.272)	-0.335 (0.434)
Information access capability	1.824** (0.811)	1.833* (0.940)	1.303 (0.991)	0.365 (1.096)	-0.503 (1.444)
Social capital networks	0.274 (0.470)	0.131 (0.359)	0.336 (0.401)	0.474 (0.531)	1.434 (0.634)
Education	0.575 (0.422)	0.434** (0.202)	0.350 (0.343)	0.304 (0.324)	-0.461 (0.599)
Non-agricultural income	0.056 (0.076)	-0.005 (0.060)	-0.066 (0.080)	-0.012 (0.116)	-0.001 (0.193)
Town	Control	Control	Control	Control	Control
Constant	-0.641 (1.401)	-0.525 (2.291)	0.412 (2.222)	0.772 (3.094)	-1.249 (7.216)
Obs	444	444	444	444	444
R^2^	0.215	0.283	0.364	0.434	0.476

The figures in parentheses represent robust standard errors; ***, ** and * denote significance levels of 1%, 5% and 10%, respectively.

This study employs quantile regression at the 10th, 25th, 50th, 75th, and 90th percentiles to examine the heterogeneous effects of variety renewal on grape unit price. The results are reported in **Table 7**. To determine whether there were significant differences in the quantile regression results across different quantiles, this study conducted a Wald test for joint significance of the quantile regression process. The results showed an F-value of 1.94 and a p-value of 0.052, indicating that the effect of variety renewal on unit price differed at the aforementioned quantile points, and that quantile regression was appropriate. For farmers adopting full renewal, the unit price of grapes increases as grape quality improves, except among farmers in the low-quality output group, for whom the estimated effect is not statistically significant. For farmers adopting partial renewal, the unit price does not show significant fluctuations. These results suggest that, in fresh table grape production, high-standard growers are better able to implement standardized cultivation, grading, sorting, and quality control. Such management capability helps maintain the quality advantages of new varieties, including appearance, taste, and market consistency. In addition, contiguous or concentrated planting may improve the uniformity of fruit appearance, which is consistent with the pricing logic of the high-end grape market. By contrast, under partial renewal, the simultaneous cultivation of old and new varieties may leave the overall product structure largely unchanged, resulting in no significant improvement in unit price. Ordinary smallholders generally have limited input capability and relatively low output levels, making it difficult for them to translate the quality advantages of new varieties into higher unit prices. For farmers at the medium level, input costs are relatively high. Those adopting full renewal can concentrate resources on the cultivation and management of new varieties, thereby ensuring the quality of new grapes and obtaining higher unit prices as level improves. However, those adopting partial renewal may face cost pressures and management difficulties associated with mixed cultivation, resulting in no significant change in unit price. High-standard growers can use economies of scale, standardized management, and quality control to reduce unit costs and improve the overall quality of new grape varieties, thereby achieving a more pronounced increase in unit price.

**Table 7:** Quantile results on the effect of variety renewal on grape unit price.

Variables	Grape unit price				
	10th percentile (1)	25th percentile (2)	50th percentile (3)	75th percentile (4)	90th percentile (5)
Complete variety renewal	0.814 (0.945)	2.926*** (0.774)	3.889*** (0.703)	5.259*** (0.736)	6.014*** (0.890)
Partial variety renewal	0.283 (0.209)	0.152 (0.146)	0.244 (0.187)	0.118 (0.292)	0.216 (0.322)
Planted area	0.021 (0.018)	0.006 (0.017)	0.006 (0.016)	-0.001 (0.047)	-0.018 (0.043)
Input cost	-0.001 (0.026)	-0.001 (0.016)	-0.001 (0.023)	-0.001 (0.011)	-0.002 (0.006)
Greenhouse	0.200 (0.152)	0.351*** (0.127)	0.644*** (0.166)	1.688*** (0.370)	2.061*** (0.669)
Training	-0.008 (0.027)	-0.005 (0.027)	-0.004 (0.035)	-0.002 (0.041)	-0.001 (0.090)
Online sales capability	-0.083 (0.076)	-0.058 (0.056)	-0.125* (0.069)	-0.072 (0.081)	-0.063 (0.162)
Information access capability	0.310 (0.278)	0.494** (0.208)	0.340** (0.146)	0.019 (0.407)	0.173 (5.718)
Social capital networks	-0.040 (0.155)	0.017 (0.173)	0.192 (0.139)	0.114 (0.179)	0.278 (0.217)
Education	0.214** (0.096)	0.019 (0.097)	-0.071 (0.089)	-0.083 (0.157)	0.033 (0.278)
Non-agricultural income	-0.003 (0.021)	-0.005 (0.016)	0.011 (0.020)	0.010 (0.033)	-0.012 (0.033)
Town	Control	Control	Control	Control	Control
Constant	1.587 (1.122)	1.986** (0827)	1.869** (0.735)	2.501** (1.191)	3.800* (2.145)
Obs	443	443	443	443	443
R^2^	0.101	0.155	0.228	0.341	0.423

The figures in parentheses represent robust standard errors; ***, ** and * denote significance levels of 1%, 5% and 10%, respectively.

Based on the comprehensive quantile regression results, the effects of variety renewal on grape yield, revenue, and unit price exhibit substantial type heterogeneity and renewal patterns. In terms of yield, full renewal is associated with a significant decline in yield among farmers with medium or lower yield levels, with the decline being larger for farmers with lower yield levels. By contrast, farmers with high yield levels do not experience a statistically significant decline in yield. Partial renewal leads to a significant yield decline mainly among farmers with medium yield levels, suggesting a bottleneck in their capability to adapt to the renewal process. In terms of grape revenue, full renewal significantly increases grape revenue among farmers with medium or higher revenue levels, and the revenue-enhancing effect becomes stronger as the revenue level increases. However, the income gains for farmers with low revenue levels are not statistically significant. By contrast, partial renewal reduces income among farmers with medium and relatively high revenue levels, reflecting the combined effects of higher costs and insufficient price premiums under mixed cultivation. In terms of unit price, full renewal is associated with higher unit prices as grape quality improves, mainly because high-standard growers are better able to implement standardized production and quality control. Ordinary smallholders, however, have difficulty translating the quality advantages of new varieties into price premiums. Partial renewal, which involves the simultaneous cultivation of old and new varieties, does not generate significant changes in unit price. From the perspective of cost–benefit heterogeneity, farmers with high production capacity and high revenue levels can achieve stable yield, higher unit prices, and increased income through full renewal by relying on cost sharing, standardized production, and quality premiums. Farmers with medium production capacity and medium revenue levels face fragmented management, relatively high costs, and pressure from yield decline under both renewal patterns, making them the most vulnerable group during the renewal process. Farmers with low production capacity and low revenue levels have weaker risk-bearing capability; full renewal entails greater adjustment risks, whereas partial renewal appears relatively stable. Overall, the economic effects of grape variety renewal are subject to clear scale thresholds. Full renewal appears more suitable for farmers with high production capacity and high revenue levels, whereas partial renewal may generate less favorable outcomes for farmers with medium production capacity and medium revenue levels. Therefore, policies promoting variety renewal should fully consider the compatibility between farmer types and renewal pattern.

The heterogeneity of variety renewal observed in the Gaizhou grape industry may provide transferable insights for the iterative upgrading of other perennial fruit industries. Compared with annual crops, perennial fruit trees generally share several industry-specific characteristics, including long biological growth cycles, high initial establishment costs, substantial sunk costs associated with variety renewal, and difficulties in managing mixed stands of old and new cultivars. These characteristics are closely aligned with the transformation logic identified in the grape industry. Therefore, the scale heterogeneity and differentiated renewal patterns revealed in this study can offer practical guidance for the upgrading of perennial fruit varieties more broadly. From the perspective of common patterns, when full variety renewal is implemented, large-scale operators are more likely to benefit from concentrated land holdings, mature production technologies, stronger standardized management capability, and the ability to spread agricultural input and management costs. These advantages enable them to adapt more rapidly to the growth requirements of new varieties, achieve quality upgrading and market premiums, and ultimately realize the transformation goals of stable production and income growth. By contrast, small- and medium-scale operators often face fragmented planting structures, insufficient refined orchard management capability, weaker technical capabilities, and a lack of scale-based premium advantages. As a result, they are more vulnerable to yield fluctuations, rising production costs, and lower-than-expected returns during full variety renewal. In addition, for perennial fruit crops, partial renewal may give rise to problems such as cultivar mix-ups, inconsistent tree ages, and non-uniform management standards. These problems can lead to unstable fruit quality, weaken the formation of brand premiums, and ultimately limit profit growth. This represents a common challenge in the partial renewal of many perennial fruit varieties. In practical implementation, full replacement and partial improvement strategies should therefore be applied in a differentiated manner according to farmers’ scale, technical capability, and risk-bearing ability. More targeted policy support, technical training, and market services should be provided to vulnerable small- and medium-scale operators so that variety renewal can contribute to the overall improvement of both the economic and social benefits of the fruit industry.

### 3.5 Mechanism analysis

Previous results indicate that the effects of variety renewal on yield and income differ across farmers of different scales, which may affect the stability of regional public brand value. One important mechanism underlying the divergence in yield and income is the change in production inputs and input-related costs associated with variety renewal. On the one hand, according to classical firm theory, rising input requirements and production costs may constrain output by increasing farmers’ production burden. These additional costs may arise from technical training, additional labor requirements, and increased production inputs during the renewal process. In the production practices of Gaizhou’s grape industry, changes in observable inputs, such as chemical fertilizers and farmyard manure, can be measured more directly, and data on these inputs are relatively accessible. Therefore, they provide useful proxies for changes in production inputs and input-related costs. On the other hand, production inputs may also affect product quality, which is closely related to grape unit price and, in turn, farmers’ income. In the context of table grapes, sweetness, grape berry bloom (the natural epicuticular wax coating on grape berries) and bunch shape are important dimensions of product quality. Among these, grape sweetness is closely related to fertilizer application, allowing this study to examine whether input changes associated with variety renewal affect product quality. Accordingly, this study proposes a mechanism through which variety renewal influences the differentiation of grape yield and income: variety renewal changes production inputs and input-related costs; these input changes affect crop output and product quality; product quality is associated with the farm-gate purchase price; and the farm-gate price further affects farmers’ income. To examine this mechanism, this study uses chemical fertilizer application rates and farmyard manure application rates as observable proxies for changes in production inputs and input-related costs. Regression analyses are then conducted to examine the empirical relationships among variety renewal, fertilizer application, and grape sweetness. According to the theoretical analysis, the relationships between input costs and yield, as well as between product quality and unit price, have been established. Therefore, this section mainly focuses on verifying the following two aspects.

First, this study examines the effects of variety renewal on the application rates of chemical fertilizers and farmyard manure. Based on the literature and the actual conditions of grape cultivation (Eba and Bashargo, 2014; Waithaka et al., 2007), the study controls for variables such as age, health status, education, planted area, growing period,number of household agricultural laborers, machinery and equipment, and greenhouses. The application rates of chemical fertilizers and farmyard manure were derived from the survey questionnaire items “How many tons of chemical fertilizer were used for grape cultivation?” and “How many tons of farmyard manure were used?”, respectively. These were converted to equivalent jin (Chinese weight units) and divided by “planted area” to obtain the application rate per mu. The results of the regression analysis are shown in **Table 8**. As shown in the table, the effects of variety renewal on chemical fertilizer and farmyard manure application rates passed the 10% and 5% significance levels, respectively. Compared to farmers who did not adopt new varieties, those who did increased their chemical fertilizer application by approximately 87 jin per mu and their farmyard manure application by approximately 508 jin per mu. In practical terms, this translates to an increase of one bag of chemical fertilizer and one cartload of farmyard manure per mu, which generally aligns with actual conditions. This indicates that variety renewal leads to additional fertilizer costs.

**Table 8:** Effects of variety renewal on fertilizer and manure application rates.

Variables	Chemical fertilizer (1)	Farmyard manure (2)
Variety renewal	86.881* (51.509)	508.357** (212.340)
Age	-0.767 (2.549)	-6.818 (10.508)
Health status	53.007 (43.136)	241.552 (177.823)
Education	8.975 (32.037)	162.259 (132.071)
Planted area	-12.737** (5.023)	-60.462*** (20.710)
Growing period	0.700 (2.466)	-2.711 (0.166)
Number of agricultural workers	28.639 (24.982)	19.384 (102.986)
Agricultural machinery	-29.441 (81.939)	412.255 (337.785)
Greenhouse	25.054 (54.023)	-56.929 (222.705)
Town	Control	Control
Constant	409.442 (252.901)	1263.528 (1042.556)
Obs	444	444
R^2^	0.066	0.054

The figures in parentheses represent robust standard errors; ***, ** and * denote significance levels of 1%, 5% and 10%, respectively.

Second, this study examines the effects of chemical fertilizer and farmyard manure application rates on grape sweetness. During the field survey, the research team randomly selected one bunch of grapes from each respondent’s vineyard and measured its sweetness using a refractometer. The average sweetness of the surveyed grape samples in Gaizhou City was 16.80, with a standard deviation of 1.89, indicating a relatively high level of sweetness. Planting area, years of cultivation experience, machinery and equipment, training participation, greenhouse use, and bagging techniques were included as control variables, as these factors may be closely related to grape sweetness in production practice. Regression analyses were conducted using log-transformed grape sweetness as the dependent variable and chemical fertilizer and farmyard manure application rates as the key explanatory variables. The results are shown in **Table 9**, the effects of chemical fertilizer and farmyard manure application rates on grape sweetness were statistically significant at the 10% and 1% levels, respectively. Specifically, a one-unit increase in chemical fertilizer application was associated with an approximately 1.4% increase in grape sweetness, while a one-unit increase in farmyard manure application was associated with an approximately 3.8% increase. These results suggest that increased input use may contribute to improvements in grape quality, thereby helping to raise the farm-gate purchase price and improve farmers’ income.

**Table 9:** Effects of chemical fertilizer and farmyard manure application rates on grape sweetness.

Variables	Grape sweetness (1)	Grape sweetness (2)
Chemical fertilizer	0.014* (0.008)	
Farmyard manure		0.038*** (0.013)
Planted area	-0.001 (0.001)	-0.002* (0.001)
Growing period	0.001* (0.000)	0.001 (0.001)
Agricultural machinery	0.014 (0.015)	0.026 (0.016)
Training	-0.000*** (0.000)	-0.000*** (0.000)
Greenhouse	0.022** (0.010)	0.018** (0.011)
Bagging techniques	0.022* (0.012)	0.022* (0.013)
Constant	2.766*** (0.018)	2.767*** (0.207)
Obs	440	374
R^2^	0.042	0.048

The figures in parentheses represent robust standard errors; ***, ** and * denote significance levels of 1%, 5% and 10%, respectively.

Taken together, the results of these two mechanism tests suggest that variety renewal is associated with changes in production inputs and input-related costs. On the one hand, according to producer theory, rising input requirements and production costs may constrain yield. On the other hand, increased input use may improve grape sweetness and product quality, thereby increasing unit prices and affecting farmers’ income. Given the relatively inelastic demand for table grapes, the increase in unit price may offset the decline in yield, leading to an overall increase in farmers’ total income. These findings indicate that input and cost changes constitute an important mechanism through which variety renewal affects yield changes and income differentiation.

## 4 Further discussion

Based on the preceding analysis, the decline in yield resulting from variety renewal is a key factor limiting the brand effect. This study explores potential measures to mitigate this decline across five dimensions, including training, information access capability, online sales capability, social learning, and social capital networks. A model was constructed to analyze the moderating effects of these five dimensions on the impact of variety renewal on grape yield. The specific model is as follows.

(3) $$y_{1}=\alpha_{i}+\;\beta_{i}x+\delta m_{i}+\gamma_{i}xm_{i}+\theta_{i}z_{1}+\epsilon_{i}$$

Here as shown in Equation (3), y_1_, x and z_1_ are the same as in the previous section, representing grape yield, variety rotation, and control variables, respectively. m_i_ is the moderating variable; to distinguish it from the sequence of Arabic numerals used earlier, we set i =a, b, c, d, e, representing, in order, training, information access capability, online sales capability, social learning, and social capital networks. ϵ_i_ represents the random error term. The study focuses on the coefficient γ_i_. To mitigate the risk of multicollinearity, this study mean-centered the variables used to construct the interaction terms. The regression results are shown in **Table 10**. Subsequently, to further examine differences across farm scales, farmers were classified into small-, medium-, and large-scale groups according to their grape cultivation area. According to the Guidelines for the Certification of Family Farms in Liaoning Province, fruit farming operations with 20 mu or more of open-field land are considered to have reached a certain operational scale. However, given the actual conditions of grape cultivation in Gaizhou City, only a relatively small proportion of farmers have reached this scale, and most farmers operate less than 20 mu. Ordinary grape growers who rely on family-contracted land for their livelihood account for a large proportion, and their contracted land averages about 5 mu, a figure closely related to household size. The number of grape growers cultivating more than 10 mu is relatively small, and these farmers usually expand their planted area through land transfers. Therefore, this study classifies planting scales based on the actual conditions of grape cultivation in Gaizhou City, farmers cultivating less than 5 mu of grapes were classified as small-scale farmers, those cultivating 5 mu or more but less than 10 mu were classified as medium-scale farmers, and those cultivating 10 mu or more were classified as large-scale farmers. To examine the heterogeneous adjustment effects across these groups, this study conducted subsample regression analyses for the five moderating dimensions. To maintain the readability of the main text, the results are reported in Appendices 2-6.

**Table 10:** Heterogeneity results.

Variables	Grape yield (1)	Grape yield (2)	Grape yield (3)	Grape yield (4)	Grape yield (5)
Variety renewal	-0.452*** (0.171)	-0.392** (0.168)	-0.489*** (0.174)	-0.454*** (0.169)	-0.394** (0.169)
Training	-0.088** (0.043)	–	–	–	–
Variety renewal*Training ( $\gamma_{a}$)	0.089** (0.043)	–	–	–	–
Information access capability	–	-0.000 (0.000)	–	–	–
Variety renewal*Information access capability ( $\gamma_{b}$)	–	0.001** (0.000)	–	–	–
Online sales capability	–	–	-0.130* (0.072)	–	–
Variety renewal*Online sales capability ( $\gamma_{c}$)	–	–	0.234** (0.117)	–	–
Social learning	–	–	–	0.000 (0.001)	–
Variety renewal*Social learning ( $\gamma_{d}$)	–	–	–	0.025** (0.012)	–
Social capital networks	–	–	–	–	-0.082** (0.041)
Variety renewal*Social capital networks ( $\gamma_{e}$)	–	–	–	–	0.112* (0.061)
Planted area	0.295*** (0.031)	0.295*** (0.031)	0.299*** (0.031)	0.296*** (0.030)	0.294*** (0.031)
Input cost	0.000*** (0.000)	-0.000 (0.000)	-0.000 (0.000)	-0.000 (0.000)	-0.000 (0.000)
Greenhouse	-0.352* (0.192)	-0.333* (0.192)	-0.399** (0.187)	-0.332* (0.191)	-0.328* (0.191)
Town	Control	Control	Control	Control	Control
Constant	1.136*** (0.393)	1.056*** (0.393)	1.107*** (0.398)	1.015** (0.391)	1.064*** (0.389)
Obs	443	443	443	443	443
R2	0.476	0.473	0.478	0.475	0.475

The figures in parentheses represent robust standard errors; ***, ** and * denote significance levels of 1%, 5% and 10%, respectively.

### 4.1 Training

Training helps improve farmers’ technical skills, which in turn affects yield (Wonde et al., 2022). In this study, “number of training sessions attended per year” is used to reflect the frequency with which farmers receive training related to grape cultivation. As shown in Column 1 of **Table 10**, the moderation effect ${\text{γ}}_{\text{a}}$was positive and passed the 5% significance level, and statistically significant at the 5% level, indicating that, under the grape production conditions in Gaizhou, training plays a significant buffering role in mitigating the adverse effect of variety renewal on yield. Since most grapes in Gaizhou are produced for fresh consumption, new varieties impose higher technical requirements in terms of local adaptation, water and fertilizer management, vine training, and pest and disease control. A lack of management experience may therefore lead to yield losses during the renewal process. By providing practical and locally applicable knowledge, including the physiological characteristics of new varieties, scientific water and fertilizer management, green pest control, and pruning techniques, training can improve growers’ technical knowledge and field-management skills, thereby reducing production losses caused by technical gaps. From the perspective of production function theory, training increases farmers’ human capital and improves the efficiency of factor allocation during the initial stage of variety renewal, thus helping to mitigate the short-term negative effect of variety renewal on yield.

The subsample regression results further show that the moderating effect of training varies across farm-size groups. Specifically, training has a statistically significant buffering effect on yield decline only among small-scale farmers, whereas its effect is not statistically significant among medium- and large-scale farmers. Small-scale grape growers in Gaizhou generally face fragmented landholdings, limited access to technical resources, and weaker adaptability to new varieties. As a result, they rely more heavily on standardized technical training, and the marginal benefits of training are stronger for this group. By contrast, medium- and large-scale farmers often have more stable management experience and stronger investment capability, leaving relatively limited room for additional improvement through training. These findings suggest that training programs for Gaizhou’s grape industry should be more targeted, with particular emphasis on technical training for small-scale farmers during the adoption of new varieties.

### 4.2 Information access capability

Information access capability is one of the key factors driving the improvement of total factor productivity in agricultural production (Mwalupaso et al., 2019). This study uses the frequency with which farmers use mobile phones and other internet-based tools to obtain grape production-related information as a proxy for information access capability, and examines its moderating effect on the relationship between variety renewal and grape yield. As shown in Column 2 of **Table 10**, the coefficient of the interaction term between variety renewal and information access capability is positive and statistically significant at the 5% level. This suggests that, under the production conditions of Gaizhou’s grape industry, information access capability helps mitigate the yield losses associated with variety renewal. Internet-based information channels can provide farmers with knowledge about the cultivation and management of new varieties, thereby partially compensating for technical gaps after variety renewal and reducing the production risks associated with the renewal process. Overall, the more frequently farmers obtain information through internet-based tools, the stronger the supporting role of digital information access in controlling yield-decline risks during variety renewal.

The subsample regression results further reveal scale-dependent heterogeneity in this moderatingeffect, as reported in Appendix Table 3. Information access capability helps mitigate yield declines among small-scale grape growers, has the opposite effect among medium-scale growers, and has no statistically significant effect among large-scale growers. Small-scale farmers often lack stable offline channels for agricultural technical guidance, and their production decisions rely heavily on fragmented experience. Therefore, formal online agricultural information can directly help fill their technical gaps. Medium-scale farmers, however, are in a transitional stage toward larger-scale operation. For this group, the large volume of fragmented and uneven-quality online information may increase decision-making uncertainty and disrupt standardized field management after variety renewal. Large-scale growers usually have more mature production systems and stronger technical capability, so the marginal effect of mobile internet-based information on their production decisions is relatively limited. These findings suggest that digital agricultural support policies for Gaizhou’s grape industry should move beyond a uniform information-provision model and develop tiered digital information services tailored to growers of different scales, thereby improving the effectiveness of internet-based information tools in supporting grape variety renewal.

### 4.3 Online sales capability

This study uses “Have you ever sold your family’s grapes via WeChat, Weibo, QQ, or similar platforms?” as a proxy for online sales participation and digital marketing capability. This indicator reflects farmers’ adoption of new distribution channels and their ability to engage in online product marketing. The moderated regression results are reported in Column 3 of **Table 10**. The coefficient of the interaction term between variety renewal and online sales capability is positive and statistically significant at the 5% level. This suggests that online sales helps mitigate yield losses associated with variety renewal. In the context of Gaizhou’s grape industry, online social sales channels can reduce farmers’ dependence on traditional buyers and alleviate the constraints of a single-channel marketing structure. These channels may also help reduce sales pressure and price suppression during the peak grape harvest season. By buffering market-side risks and improving farmers’ sales expectations, online sales can support farmers in maintaining production inputs and field management during the variety renewal process, thereby reducing yield losses caused by short-term adaptation difficulties.

The subsample regression results further show that the moderating effect of online sales differsacross farm-size groups, as reported in Appendix Table 4. For large-scale growers, a relatively mature online sales system can help buffer the production uncertainty associated with variety renewal, and the benefits generated on the marketing side can be transmitted back to production decisions. Therefore, the buffering effect of online sales is statistically significant for this group. For small- and medium-scale farmers, however, online sales do not exert a statistically significant moderating effect on the negative yield impact of variety renewal. Accordingly, the development of digital e-commerce in Gaizhou’s grape industry should first strengthen the independent online sales channels of large-scale farmers, while also providing differentiated e-commerce support for small- and medium-scale farmers. This would help better leverage the risk-buffering value of online sales during grape variety renewal.

### 4.4 Social learning

From an input-output perspective, social learning can help farmers efficiently access new agricultural production technologies and market information (Xue et al., 2022), thereby enhancing human capital and, in turn, influencing yields. This section analyzes the moderating effect of this indicator on the impact of variety renewal on grape yield in the short term. Social learning is measured based on the question: “In the past year, how many times have growers proactively learned about grape production and sales through mobile phones, computers, books, and other sources?” As shown in Column 4 of **Table 10**, social learning positively moderates the relationship between variety renewal and grape yield, with a positive moderating effect that passes the 5% significance level. Given the production conditions of Gaizhou’s grape industry, farmers’ engagement in social learning can help address technical gaps in the cultivation of new varieties and improve their human capital. Therefore, stronger social learning capability may weaken the short-term adverse effect of variety renewal on grape yield.

The subsample regression results reported in Appendix Table 5 indicate that the moderating effect of social learning varies across farm-size groups. For large-scale grape growers, their larger production scale and relatively stronger technical capability enable them to translate learning inputs more efficiently into production performance. As a result, social learning can help offset yield fluctuations associated with variety renewal, and its negative moderating effect is statistically significant for this group. In contrast, small- and medium-scale farmers generally have more limited production scale, weaker technical absorptive capability, and fewer complementary resources. Consequently, social learning does not show a statistically significant moderating effect among these groups. For large-scale farmers undertaking a complete variety renewal strong social learning is particularly crucial, as they face greater technical transition and management challenges; social learning helps them establish production management systems adapted to new varieties more quickly.

### 4.5 Social capital networks

This section analyzes the moderating effect of social capital networks on the impact of short-term variety renewal on grape yield. Social capital networks evaluates the stock and quality of social capital, which can reduce costs through resource integration and mitigate issues such as technological mismatches that may arise from variety renewal. The study measures the height of social capital among grape growers by assessing “how many relatives and friends of the survey respondents work in government.” As shown in Column 5 of **Table 10**, the height of social capital effectively promotes grape yield, with a positive moderatingeffect that passes the 10% significance level. This implies that when farmers possess a higher levelof social capital, the adverse impact of variety renewal on yield is mitigated to some extent. The higher the level of social capital networks, the weaker the short-term negative impact of variety renewal on grape yields. The results of the subgroup regression analysis (see Appendix Table 6) show that social capital does not have a significant moderating effect for either large-scale farmers or small- and medium-scale farmers. This indicates that the moderating effect is reflected only in the differences between groups in the full sample, rather than in the within-group effects. At the same time, the reduction in sample size after grouping may also have affected the test results to some extent.

## 5 Conclusions and recommendations

### 5.1 Conclusions

In the short term, variety renewal is associated with a significant decline in grape yield, a significant increase in farmers’ total income, and a significant rise in grape unit price. Specifically, farmers who adopted variety renewal experienced an annual yield reduction of approximately 3,800 jin, while their total annual income increased by about 16,000 yuan. The unit price of Shine Muscat was significantly higher than that of Kyoho, with the farm-gate purchase price increasing by approximately 1.57 yuan per jin. After controlling for planting scale, facilities and equipment, and farmer characteristics, farmers who adopted variety renewal achieved higher total income than those who continued to cultivate traditional Kyoho grapes, although their yield declined to some extent. This finding is consistent with the economic logic that grape growers adopt new varieties to pursue higher income through price premiums.

The quantile regression results further indicate that the effects of variety renewal on grape yield, revenue, and unit price exhibit substantial heterogeneity across renewal patterns and different points of the outcome distributions. In terms of yield, full renewal leads to a significant yield decline among farmers with medium or lower yield levels, with the decline being larger for farmers at lower yield quantiles. By contrast, farmers with higher yield levels do not experience a statistically significant decline in yield. Partial renewal leads to a significant yield decline mainly among farmers with medium and near-medium yield levels, suggesting a bottleneck in their capability to adapt to the renewal process. In terms of revenue, full renewal significantly increases revenue among farmers with medium or higher revenue levels, and the revenue-enhancing effect becomes stronger as the revenue level increases. However, the revenue gains for farmers with lower revenue levels are not statistically significant. Partial renewal, by contrast, reduces revenue among farmers with medium and relatively higher revenue levels, reflecting the combined effects of higher costs and insufficient price premiums under mixed cultivation. In terms of unit price, full renewal is associated with higher unit prices as grape quality and market position improve, mainly because farmers with higher-quality output are better able to implement standardized production and quality control. Farmers in the low-quality output group, however, have difficulty translating the quality advantages of new varieties into price premiums. Partial renewal, which involves the simultaneous cultivation of old and new varieties, does not generate significant changes in unit price. From the perspective of outcome-level heterogeneity, farmers with higher production capacity, stronger revenue performance, and better quality-control capability can achieve relatively stable yield, higher unit prices, and increased revenue through full renewal by relying on cost sharing, standardized production, and quality premiums. Farmers at medium outcome levels face fragmented management, relatively high costs, and pressure from yield decline under both renewal patterns, making them more vulnerable during the renewal process. Farmers at lower outcome levels have weaker risk-bearing capability; full renewal entails greater adjustment risks, whereas partial renewal appears relatively stable. Overall, the economic effects of grape variety renewal are subject to clear production-capacity and market-performance thresholds. Full renewal appears more suitable for farmers with higher yield levels, stronger revenue performance, and better quality-control capability, whereas partial renewal may generate less favorable outcomes for farmers at medium outcome levels. Therefore, policies promoting variety renewal should fully consider the compatibility between renewal patterns and farmers’ positions in the yield, revenue, and unit price distributions.

The mechanism analysis shows that changes in production inputs and input-related costs constitute an important channel through which variety renewal affects yield and income differentiation. On the one hand, variety renewal increases production and management requirements, which may raise input-related costs and constrain yield in the short term. On the other hand, increased input use may improve grape sweetness and product quality, thereby raising unit prices. The resulting price premium can offset part of the yield loss and contribute to higher total income. These findings suggest that input and cost changes help explain both the short-term yield decline and the income-enhancing effect associated with variety renewal.

Furthermore, this study examines five dimensions that may mitigate yield losses during the renewal process: farmer training, information access, online sales participation, social learning, and social capital networks. The results show that increasing the frequency of technical training, improving farmers’ information access capability, expanding multi-channel online sales, enhancing social learning capability, and strengthening social capital networks can help mitigate the negative effect of variety renewal on grape yield. The subsample regression results further indicate that training and information access capability have stronger buffering effects among small-scale farmers, and online sales and social learning help mitigate short-term yield risks among large-scale farmers.

### 5.2 Recommendations

First, differentiated support policies should be adopted to facilitate variety renewal across farm-size groups. For agricultural operators adopting full renewal, support measures should follow the principle of scale-appropriate intervention. Large-scale farmers are better positioned to adopt full renewal because they can reduce input costs through bulk purchasing, standardized management, and centralized production. For this group, policy support should focus on improving standardized production services, strengthening quality control, and encouraging qualified growers to participate in the use and protection of the geographical indication (GI) brand, thereby further enhancing stable yield, higher income, and quality premiums. Medium-scale farmers face greater challenges during full renewal, including yield decline, high management costs, and limited risk-bearing capability, making them a vulnerable group during the renewal process. Transitional subsidies for yield stabilization and low-interest special loans should be introduced to relieve financial pressure and provide sufficient adjustment space. Small-scale farmers should be discouraged from blindly adopting full renewal. Instead, they should be encouraged to form mutual-aid groups or participate in cooperatives, so that unified management, shared services, and centralized marketing can help reduce operational costs. For farmers adopting partial renewal, especially medium- and large-scale farmers, targeted guidance on cost control should be provided. A graded procurement system for grapes should also be established to address the problems of high costs and insufficient price premiums under mixed cultivation. For small-scale farmers, transitional income support and risk protection should be strengthened to help them pass through the variety renewal period more smoothly.

Second, efforts should be anchored in geographical indication protection to build a full-chain brand management system. Relying on the regional brand advantage of the Gaizhou Grape GI, a brand management system characterized by unified standards, strict quality control, and full traceability should be established. This system should be centered on the diversified product structure generated by variety renewal. Based on the growth characteristics and quality requirements of different varieties, such as Kyoho and Shine Muscat, localized technical specifications should be formulated. These specifications should clearly define key requirements for seedling selection, field management, water and fertilizer use, pesticide application, fruit shape, and sugar content. Brand access thresholds should be established, and the right to use the GI label should be granted only to growers who meet the required production and quality standards. In addition, a unified county-level traceability platform for agricultural products should be developed. Traceability codes should be assigned according to farm scale, variety type, and production entity, with comprehensive records of field management, quality testing results, and sales channels. GI products should be required to display traceability codes, and traceability records and quality ratings should be linked to policy support and brand-use eligibility. Such full-chain supervision can help protect the reputation of the regional public brand and consolidate the foundation for a “quality-for-price” development model.

Third, multidimensional capability building should be provided through a tiered approach to mitigate the risk of yield decline during the renewal period. To alleviate the short-term yield losses associated with variety renewal, targeted support should be provided according to the needs of farmers of different scales through five pathways: technical training, information access, online sales, social learning, and social capital networks. For small-scale farmers, technical training should focus on hands-on field instruction. Regular on-site guidance should be provided on vine management, water and fertilizer use, pest and disease control, and post-renewal maintenance. At the same time, village-level information service stations should be improved to disseminate timely information on production techniques, market prices, pest and disease warnings, and agricultural support policies, thereby enhancing farmers’ capability to use information-based tools. For large-scale farmers, support should focus on developing diversified online sales channels, including e-commerce platforms, social media marketing, and live-streaming sales. These channels should be complemented by cold-chain storage and logistics facilities to reduce post-harvest losses and strengthen market resilience. In addition, demonstration orchards and regular exchange meetings among large-scale farmers should be established to create social learning platforms, promote the rapid diffusion of advanced cultivation and management practices, and further consolidate their advantages in stable production and income growth. For medium-scale farmers, support should focus on strengthening the role of social capital networks.

**Figure 2:**
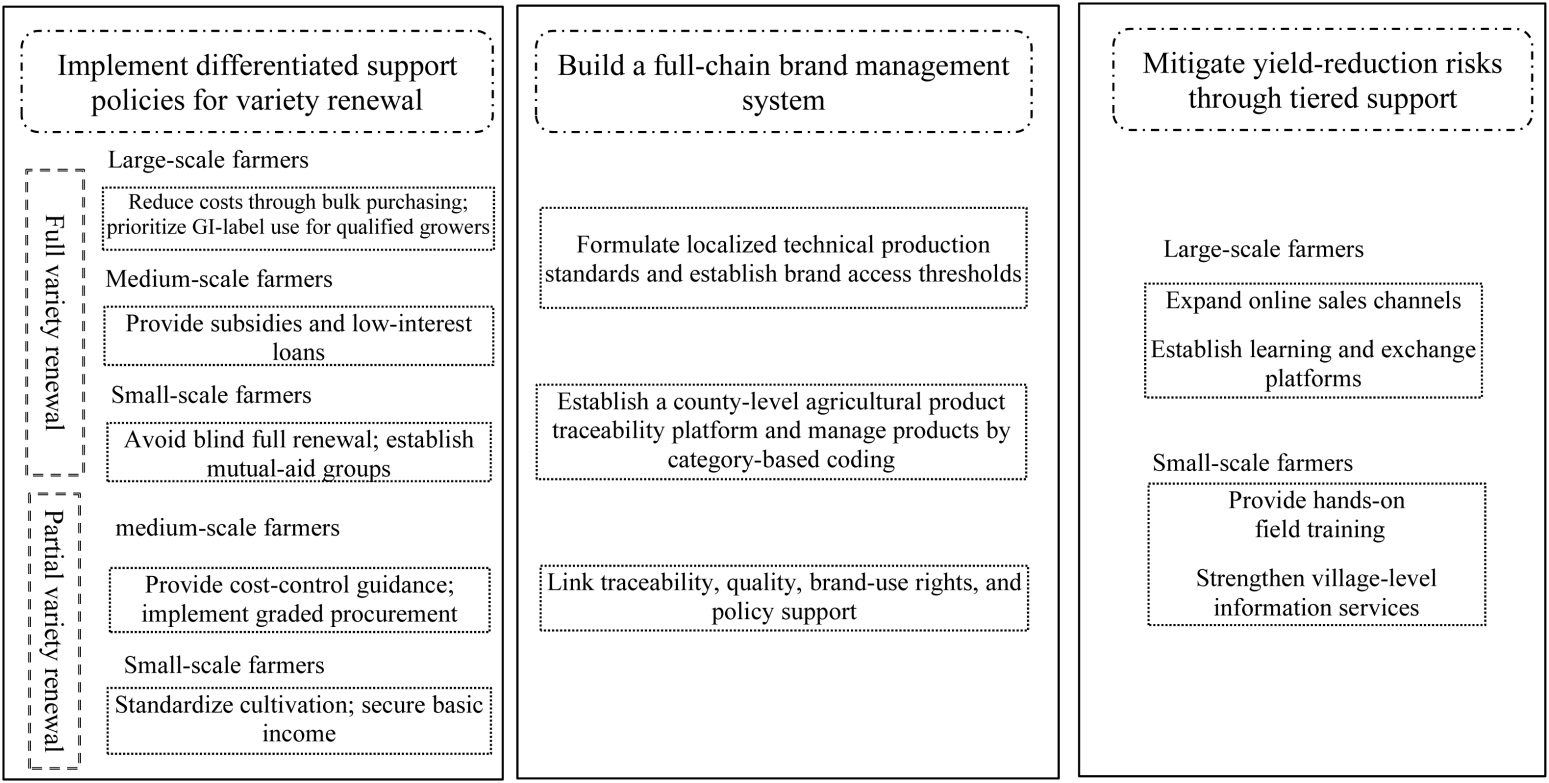
Policy recommendation process.

The policy recommendation process is presented in **Figure 2**.

## Data Availability

The original contributions presented in the study are included in the article/supplementary material. Further inquiries can be directed to the corresponding authors.

## Appendix

**Appendix Table 1:** Weak instrument test results.

First-stage regression statistics					
Grape yield					
var	R-sq	Adjusted R-sq	Partial R-sq	F	P
Variety renewal	0.284	0.274	0.029	10.949	0.001
Grape revenue					
var	R-sq	Adjusted R-sq	Partial R-sq	F	P
Variety renewal	0.289	0.271	0.028	10.864	0.001
Grape unit price					
var	R-sq	Adjusted R-sq	Partial R-sq	F	P
Variety renewal	0.290	0.272	0.028	10.692	0.001

**Appendix Table 2:** Heterogeneous effects of training on grape yield across farm-size groups.

Variables	Small-scale farmers (1)	Medium-scale farmers (2)	Large-scale farmers (3)
Variety renewal	-0.605*(0.319)	-0.441**(0.222)	-0.331(0.577)
Training	-0.082(0.070)	0.046(0.069)	-0.190(0.269)
Variety renewal*Training( $\gamma_{a}$)	0.318***(0.104)	-0.035(0.119)	0.318(0.305)
Input cost	0.234***(0.081)	0.000(0.001)	0.151***(0.051)
Greenhouse	-0.234(0.276)	0.092(0.232)	-1.236**(0.557)
Town	Control	Control	Control
Constant	0.766(0.549)	2.432***(0.456)	5.975***(1.072)
Obs	158	186	99
R2	0.109	0.096	0.257

The figures in parentheses represent robust standard errors; ***, ** and * denote significance levels of 1%, 5% and 10%, respectively.

**Appendix Table 3:** Heterogeneous effects of information access capability on grape yield across farm-size groups.

Variables	Small-scale farmers (1)	Medium-scale farmers (2)	Large-scale farmers (3)
Variety renewal	-0.911*(0.426)	0.454(0.325)	-0.223(0.606)
Information access capability	-0.000(0.000)	-0.000(0.000)	-0.049(0.085)
Variety renewal*Information access capability( $\gamma_{b}$)	0.092*(0.042)	-0.130**(0.039)	0.050(0.085)
Input cost	0.000(0.001)	0.000(0.000)	0.184***(0.020)
Greenhouse	0.088(0.111)	0.039(0.189)	-1.330*(0.627)
Town	Control	Control	Control
Constant	1.677***(0.194)	3.011***(0.161)	4.978***(0.235)
Obs	158	186	99
R2	0.025	0.068	0.221

The figures in parentheses represent robust standard errors; ***, ** and * denote significance levels of 1%, 5% and 10%, respectively.

**Appendix Table 4:** Heterogeneous effects of online sales on grape yield across farm-size groups.

Variables	Small-scale farmers (1)	Medium-scale farmers (2)	Large-scale farmers (3)
Variety renewal	-0.057(0.326)	-0.539**(0.213)	-0.635(0.543)
Online sales	0.090(0.092)	-0.235***(0.083)	-0.768***(0.283)
Variety renewal*Online sales ( $\gamma_{c}$)	-0.135(0.191)	0.181(0.162)	1.067**(0.424)
Input cost	-0.001(0.001)	0.000(0.001)	0.158***(0.050)
Greenhouse	-0.130(0.286)	-0.053(0.230)	-1.378**(0.537)
Town	Control	Control	Control
Constant	1.095**(0.568)	2.560***(0.448)	6.394***(1.027)
Obs	158	186	99
R^2^	0.055	0.134	0.310

The figures in parentheses represent robust standard errors; ***, ** and * denote significance levels of 1%, 5% and 10%, respectively.

**Appendix Table 5:** Heterogeneous effects of social learning on grape yield across farm-size groups.

Variables	Small-scale farmers (1)	Medium-scale farmers (2)	Large-scale farmers (3)
Variety renewal	-0.279(0.290)	-0.502**(0.139)	-1.152**(0.290)
Social learning	0.000(0.000)	-0.022***(0.003)	-0.317***(0.030)
Variety renewal* Social learning( $\gamma_{d}$)	0.000(0.007)	0.017(0.021)	0.369***(0.031)
Input cost	-0.001***(0.000)	0.000(0.000)	0.165***(0.023)
Greenhouse	0.103(0.110)	-0.001(0.201)	-1.446*(0.584)
Town	Control	Control	Control
Constant	1.679***(0.184)	3.107***(0.172)	6.022***(0.354)
Obs	158	186	99
R2	0.013	0.039	0.271

The figures in parentheses represent robust standard errors; ***, ** and * denote significance levels of 1%, 5% and 10%, respectively.

**Appendix Table 6:** Heterogeneous effects of social capital networks on grape yield across farm-size groups.

Variables	Small-scale farmers (1)	Medium-scale farmers (2)	Large-scale farmers (3)
Variety renewal	-0.309(0.314)	-0.477*(0.187)	0.092(0.539)
Social capital networks	-0.060(0.050)	-0.141**(0.048)	-0.002(0.116)
Variety renewal*Social capital networks( $\gamma_{e}$)	0.088(0.104)	0.123(0.065)	0.065(0.095)
Input cost	-0.001***(0.000)	0.000(0.000)	0.186***(0.022)
Greenhouse	0.120(0.124)	-0.002(0.202)	-1.344*(0.596)
Town	Control	Control	Control
Constant	1.686***(0.186)	3.062***(0.161)	4.686***(0.416)
Obs	158	186	99
R2	0.015	0.044	0.217

The figures in parentheses represent robust standard errors; ***, ** and * denote significance levels of 1%, 5% and 10%, respectively.
